# Comparison of Culture-Dependent and Culture-Independent Methods for Routine Identification of Airborne Microorganisms in Speleotherapeutic Caves

**DOI:** 10.3390/microorganisms12071427

**Published:** 2024-07-14

**Authors:** Rok Tomazin, Tjaša Cerar Kišek, Tea Janko, Tina Triglav, Katja Strašek Smrdel, Vesna Cvitković Špik, Andreja Kukec, Janez Mulec, Tadeja Matos

**Affiliations:** 1 Institute of Microbiology and Immunology, Faculty of Medicine, University of Ljubljana, Zaloška Cesta 4, SI-1000 Ljubljana, Slovenia; rok.tomazin@mf.uni-lj.si (R.T.); tina.triglav@mf.uni-lj.si (T.T.); katja.strasek@mf.uni-lj.si (K.S.S.); vesna.cvitkovic-spik@mf.uni-lj.si (V.C.Š.); 2 National Laboratory for Health, Environment and Food, Department for Public Health Microbiology, Grablovičeva Ulica 44, SI-1000 Ljubljana, Slovenia; tjasa.cerar.kisek@nlzoh.si (T.C.K.); tea.janko@nlzoh.si (T.J.); 3 Department of Public Health, Faculty of Medicine, University of Ljubljana, Zaloška Cesta 4, SI-1000 Ljubljana, Slovenia; andreja.kukec@mf.uni-lj.si; 4 Karst Research Institute, Research Centre of the Slovenian Academy of Sciences and Arts, Titov Trg 2, SI-6230 Postojna, Slovenia; janez.mulec@zrc-sazu.si; 5 UNESCO Chair on Karst Education, University of Nova Gorica, SI-5271 Vipava, Slovenia

**Keywords:** MALDI-TOF MS, OmniLog ID System, microbial identification, metagenomics, speleotherapy, cave aerobiology, nitrocellulose filters

## Abstract

The effective identification of bacterial and fungal isolates is essential for microbiological monitoring in environments like speleotherapeutic caves. This study compares MALDI-TOF MS and the OmniLog ID System, two high-throughput culture-based identification methods. MALDI-TOF MS identified 80.0% of bacterial isolates to the species level, while the OmniLog ID System identified 92.9%. However, species-level matches between the methods were only 48.8%, revealing considerable discrepancies. For discrepant results, MALDI-TOF MS matched molecular identification at the genus level in 90.5% of cases, while the OmniLog ID System matched only in 28.6%, demonstrating MALDI-TOF MS’s superiority. The OmniLog ID System had difficulties identifying genera from the order *Micrococcales*. Fungal identification success with MALDI-TOF MS was 30.6% at the species level, potentially improvable with a customised spectral library, compared to the OmniLog ID System’s 16.7%. Metagenomic approaches detected around 100 times more microbial taxa than culture-based methods, highlighting human-associated microorganisms, especially *Staphylococcus* spp. In addition to *Staphylococcus* spp. and *Micrococcus* spp. as indicators of cave anthropisation, metagenomics revealed another indicator, *Cutibacterium acnes*. This study advocates a multi-method approach combining MALDI-TOF MS, the OmniLog ID System, culture-based, and metagenomic analyses for comprehensive microbial identification. Metagenomic sampling on nitrocellulose filters provided superior read quality and microbial representation over liquid sampling, making it preferable for cave air sample collection.

## 1. Introduction

The identification of microorganisms is a cornerstone of both environmental and medical microbiology. It plays a crucial role in the understanding and management of microbial diversity, ecosystem functions, and human health [1,2]. MALDI-TOF MS is routinely used as a rapid and cost-effective method for pathogen identification in clinical settings [3,4]. However, its performance on complex environmental samples has yet to be further tested [5,6,7,8,9]. For example, in previous studies of show and speleotherapeutic caves, MALDI-TOF MS has already been successfully used in evaluating the anthropogenic impact on the cave aerobiome. With MALDI-TOF MS, we could generally identify between <50% and more than 90% of bacterial isolates—depending on the degree of cave anthropisation and the version of Bruker’s mass spectral library [5,7,10].

In addition to MALDI-TOF MS, other culture-dependent identification methods are also used to identify microorganisms [11]. These include the OmniLog ID System, which is based on a series of biochemical assays. The OmniLog ID System is not only used for identification purposes but also and especially for the metabolic characterisation of bacterial and fungal isolates [12,13,14,15], antimicrobial susceptibility testing [16,17], physiological characterisation of new microbial species, and phenomic characterisation of complex microbial communities such as biofilms [18,19,20].

Both identification methods have high-throughput capability and a broad database coverage but are limited to cultivable microorganisms. A culture-based approach alone is not sufficient to cover the whole microbial diversity and analyse complex microbial communities, as less than 2% of microorganisms can be cultured in standard laboratory conditions [21,22,23]. At this point, metagenomic analyses gain importance, as they can detect the non-cultivable part of the microbiota.

Monitoring the microbiological quality of the environment, especially in the context of human health and rehabilitation, is essential. This is particularly important in the case of speleotherapy. Speleotherapy uses the climatic conditions of caves and salt mines for the rehabilitation treatment of chronic and allergic respiratory diseases, in particular chronic obstructive pulmonary disease (COPD) and asthma, as well as some dermatological diseases such as atopic dermatitis in children [24,25,26]. The reason for the clinical improvement in these diseases lies in the microclimatic properties and the low biocontamination of the caves, which reduces the inflammatory response [24,27,28,29]. The monitoring of bioaerosols and climatic conditions in speleotherapeutic caves is therefore important both for the success of the therapy and for environmental protection [30,31,32]. The microbiological monitoring of air quality in caves is not yet standardised and uses various approaches, including traditional culture-based methods and modern genomic analyses [32]. 

The aim of this study was to compare the identification success of the two culture-based approaches and the re-identification of isolates with poor or unreliable identification using 16S rRNA gene amplicon sequencing. In addition, to obtain a complete overview and impact of patients on the airborne microbial diversity, metagenomic analysis was included in this study as well. Microbiological samples were collected from the air of the Sežana Hospital Cave in Slovenia, which is used for speleotherapy. This study fills a data gap on cave aerobiology, which is underrepresented in cave studies compared to other cave microhabitats. The cave microbiota has already been investigated metagenomically in several studies, examining limestone, soil and sediments, speleothems, and the biotechnological potential of these cave communities [33,34,35,36]. This study aimed also to give recommendations on the microbiological monitoring of air quality in speleotherapeutic caves.

## 2. Materials and Methods

The materials and methods for air sampling, cultivation, and MALDI-TOF MS identification were comprehensively detailed in our previous study on the effects of speleotherapy on cave aerobiota [10] and are only summarised here.

### 2.1. Sampling Sites

**Figure 1:**
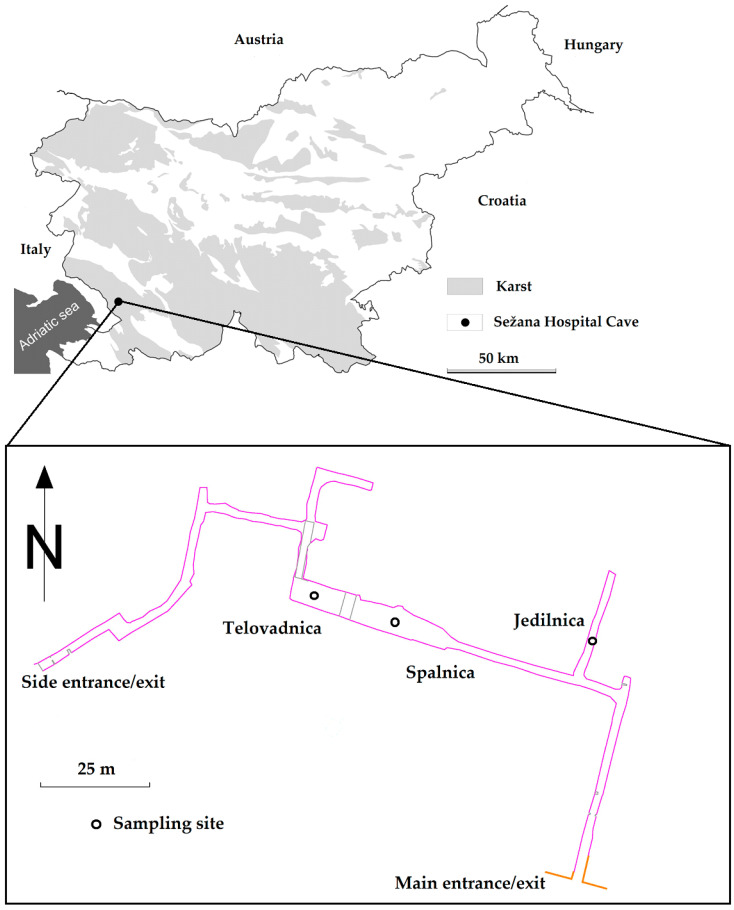
Location of the Sežana Hospital Cave in Slovenia and the sampling sites in the cave itself. The floor plan is adapted from the register of the Karst Research Institute, the Research Center of the Slovenian Academy of Sciences and Arts.

The air samples were collected in a semi-artificial cave used for speleotherapy in the Sežana Hospital, Slovenia (45°42′33.6672″ N, 13°52′25.7448″ E, 364 m a.s.l.). The hospital treats chronic respiratory diseases and has been using a semi-artificial karst cave behind the hospital for rehabilitation since 1993. This cave, which was originally dug during the Second World War, comprises a 207 m long tunnel with two entrances and a central chamber that was formerly used as a storage room (Figure 1). The main therapeutic area spans approximately 407 m³, with the total volume of the cave being approximately 1321 m³. The sampling locations were selected according to therapeutic activities: a lunch break corridor (“Jedilnica”), a meditation and education area in the centre of the chamber (“Spalnica”), and a physical activity area at the chamber’s opposite end (“Telovadnica”) (Figure 1).

### 2.2. Air Sampling

The sampling, previously detailed in a comprehensive study on the effects of speleotherapy on Sežana Hospital Cave’s aerobiota dynamics [10], involved two main phases. Firstly, air sampling began several hours before patient access to establish natural baseline conditions. After the group of patients left the cave, the second round of sampling began to assess their direct impact on the aerobiota. Sampling took place on 10 January 2023. 

Three different air samplers were used simultaneously: a VWR^®^ SAS Super DUO 360 Air Sampler (impactor) (VWR International, Radnor, PA, USA) for direct collection on contact agar plates, a Coriolis^®^µ Cyclonic Air Sampler (impinger) (Bertin Technologies, Saint Quentin en Yvelines, France) for collection in saline solution (0.9% NaCl solution), and the MAS-100 NT^®^ Air Sampler System (impactor) (Merc KGaA, Darmstadt, Germany) for collection on nitrocellulose filters with a pore size of 0.45 µm (Prat Dumas, Bourg, France). The impactor VWR^®^ SAS Super DUO 360 Air Sampler simultaneously sampled 0.5 m^3^ of air on two contact plates with different growth media: BD^TM^ Columbia Agar (Becton-Dickinson, Franklin Lakes, NJ, USA) and Sabouraud Dextrose Agar (SDA) (Oxoid Limited, Basingstoke, UK). The impinger collected airborne particles from 4.5 m^3^ of air in sterile saline solution, while the MAS-100 NT Air Sampler impacted particles from 4.0 m^3^ of air on nitrocellulose filters. Prior to sampling, all surfaces of the devices were thoroughly disinfected with 96% ethanol. 

After sampling, the RODAC plates were sealed, and the liquid samples were divided for individual microbiological analyses. The nitrocellulose filters were stored at −80 °C until further processing of the DNA isolation. The samples from the impactor VWR^®^ SAS Super DUO 360 Air Sampler and the impinger were used for culture-based analyses, while the samples from the MAS-100 NT Air Sampler and the impinger were used for metagenomics.

### 2.3. Microbial Cultivation

During air sampling with the impactor, two RODAC plates were used simultaneously on each of the two heads of the air sampler: BD^TM^ Columbia Agar with 5.0% sheep blood (BA) (Becton-Dickinson, Franklin Lakes, NJ, USA) and Sabouraud Dextrose Agar with chloramphenicol (SDA) (Oxoid Limited, Basingstoke, UK). BA and SDA were chosen to estimate and identify the cultivable fraction of airborne bacteria and fungi, respectively. Following the manufacturer’s instructions, both media were prepared and poured into the RODAC Petri dishes. The control strains *Escherichia coli* ATCC 25922 and *Staphylococcus aureus* ATCC 25923 were used for BA, while *Aspergillus brasiliensis* ATCC 16404 and *Candida albicans* ATCC 10231 served as controls for SDA.

After sampling, the sealed RODAC plates were transported to the Institute of Microbiology and Immunology, Faculty of Medicine, University of Ljubljana, Slovenia. Incubation took place at 37 °C for 48 h (BA) and at 20 °C for 7 days (SDA), with daily growth monitoring.

For impinger-based sampling, BD^TM^ Columbia Agar (Becton-Dickinson, Franklin Lakes, NJ, USA) and Sabouraud Dextrose Agar plates (SGC2) supplemented with gentamicin and chloramphenicol (bioMérieux, Marcy-l’Étoile, France) were used to determine the bacterial and fungal fractions, respectively. Liquid samples (200 µL) were evenly distributed on BA and SGC2 plates and then incubated for 48 h at 37 °C and 7 days at 20 °C, respectively. Distinct bacterial and fungal morphotypes sampled with the impactor and impinger were subjected to identification by MALDI-TOF MS and the OmniLog ID System.

Bacterial colonies on the primary selection agar media were further identified by MALDI-TOF MS and the OmniLog ID System from pure cultures after incubation on BA at 37 °C for 24 to 48 h. From distinct fungal morphotypes, pure cultures were made on SGC2 and incubated at 37 °C or 30 °C (depending on preliminary morphological identification [37,38]) for 24 to 72 h, followed by identification based on morphological characteristics, MALDI-TOF MS, and the OmniLog ID System.

### 2.4. Microbial Identification

#### 2.4.1. MALDI-TOF MS Identification

Microbial isolates from BA and SGC2 plates were identified by MALDI-TOF MS with a formic acid on-spot extraction method using the established procedures described previously [5,10]. The spectra obtained were analysed using the MALDI-TOF Biotyper^®^ (MBT) Compact HT software, with the Main Spectra Library BDAL v. 2023 for bacteria and yeasts and Filamentous Fungi v. 2023 for moulds (Bruker Daltonik, Bremen, Germany). The quality of identification was assessed using scores from 0 to 3 assigned by the manufacturer. Scores ≥ 2.00 indicated reliable species-level identification, 1.70 to 1.99 indicated genus-level identification, and scores < 1.70 were considered unidentified. Additionally, filamentous fungi were identified based on growth and morphological characteristics [37,38].

#### 2.4.2. OmniLog ID System Identification

The same microbial isolates that were subjected to MALDI-TOF MS identification were also identified using the OmniLog ID System (Biolog Inc., Hayward, CA, USA) according to the manufacturer’s instructions. This method allows the identification of bacteria, yeasts, and moulds using three different OmniLog MicroPlates: GEN III MicroPlates™ for bacteria, YT MicroPlates™ for yeasts, and FF MicroPlates™ for moulds (Biolog Inc., Hayward, CA, USA). The MicroPlates evaluate the ability of microbial isolates to metabolise up to 95 different carbon sources. In the case of the GEN III MicroPlates™, 23 chemical susceptibility tests are included in addition to 71 assimilation tests. All MicroPlates™ contained a tetrazolium redox dye, which was used to calorimetrically indicate positive reactions. The inoculation procedure was based on the original MicroPlate method according to the manufacturer’s protocol (Biolog Inc., Hayward, CA, USA). 

Prior to the OmniLog identification procedure, bacterial isolates were cultured on BA, and fungal isolates were cultured on 2% malt extract agar (MEA, Biolog Inc., Hayward, CA, USA). The microbial colonies were transferred to the test-specific inoculation fluid using a sterile wooden Biolog Streakerz™ stick to generate cell suspensions whose transmittance level was adjusted to 95–98% for bacteria and 73–77% for fungi using a turbidimeter (Biolog Inc., Hayward, CA, USA). Then, 100 µL of the cell suspension was added to each test well. The absorbance in each well of the inoculated MicroPlates was measured at 590 nm on a Biolog MicroStation™ (Biolog Inc., Hayward, CA, USA) at 8, 16, and 22 h for GEN III MicroPlates and at 24 h intervals over seven days for YT and FF MicroPlates. The results were recorded and analysed using MicroLog™ software (Biolog Inc., Hayward, CA, USA). For moulds, the Air Database (Biolog Inc., Hayward, CA, USA) was used as it contains the physiological patterns for all common airborne fungi. The results were presented as a similarity index (0.000–1.000) for all three MicroPlate types and also as the probability of correct identification (%) for the GEN III MicroPlates only, according to the manufacturer’s instructions. The identification of the fungal isolates was combined with their growth and morphological characteristics [37,38]. For GEN III MicroPlates (identification of bacteria), at least 50.0% probability after 22 h of incubation indicated a species-level identification. For YT MicroPlates (identification of yeasts), a similarity index of ≥0.75 after 24 h of incubation was considered an acceptable species identification. After 48 h or 72 h incubation, the similarity index had to be at least 0.5 to be considered acceptable. For FF MicroPlates (identification of moulds), a similarity index of ≥0.9 after 24 h of incubation was considered acceptable species identification. A similarity index of at least 0.7 after 48 h of incubation was considered acceptable. After 72 h of incubation, the similarity index had to be at least 0.65 to be considered acceptable and at least 0.6 after 96 h of incubation. 

#### 2.4.3. Molecular Identification of Bacteria

Bacterial isolates with discrepant identifications by MALDI-TOF MS and the OmniLog ID System were subjected to 16S ribosomal RNA (16S rRNA) identification. First, bacterial DNA for PCR was isolated from pure bacterial cultures using the InstaGene Matrix (Bio-Rad, Hercules, CA, USA). The partial sequence of the 16S rRNA gene was amplified using the Mastermix 16S Complete Kit (Molzym GmbH, Bremen, Germany) on the Light-Cycler^®^ 480 Instrument II Real-Time PCR System (Roche Diagnostics, Basel, Switzerland) and sequenced using the Applied Byosistems 3500 Series Genetic Analyser (Applied Bio-systems, Waltham, MA, USA), in each case according to the manufacturer’s instructions. The partial sequences of the 16S rRNA gene were compared with the public sequence database Gen-Bank using the Nucleotide BLAST programme available on the National Centre for Bio-technology Information NCBI server (Nucleotide BLAST: Searchnucleotidedatabasesus-inganucleotidequery (https://www.nih.gov/)). Sequences with a match of ≥99.0% or ≥97.0% with a database sequence were considered to belong to the same species or genus as the sequence with the highest similarity [39].

#### 2.4.4. Metagenomic and Statistical Analysis

We performed nucleic acid extraction from ¼ of each nitrocellulose filter using the AllPrep DNA/RNA Micro Kit (Qiagen, Hilden, Germany), following the manufacturer’s protocols, resulting in 50 µL eluate. Nucleic acids were extracted from 200 µL of each collection liquid in duplicate using the AllPrep PowerViral DNA/RNA Kit (Qiagen, Hilden, Germany), following the manufacturer’s protocols. DNA concentration was determined using the Qubit dsDNA HS Assay Kit (Thermo Fisher Scientific, Waltham, MA, USA) according to the manufacturer’s instructions.

Prior to automated cluster generation and sequencing, whole-genome amplification was performed with the isothermal multiple displacement amplification technique using the REPLI-G Mini Kit (Qiagen, Hilden, Germany) according to the manufacturer’s instructions. Illumina sequencing libraries were prepared using the Nextera DNA Flex Library Prep Kit (Illumina, San Diego, CA, USA) and sequenced using the NexSeq 2000 System (150 bp paired-end reads; Illumina) according to the manufacturer’s specifications. After sequencing the whole genome of the community and analysing it with Kraken 2 (Centre for Computational Biology, John Hopkins University, Baltimore, MD, USA), a fast and accurate taxonomic sequence classifier that assigns taxonomic labels to DNA sequences based on their k-mer signatures, and the Pavian R package v1.2.0, which processes the results of the Kraken 2 taxonomic classification and visualises the sequences, the results were presented in the form of the absolute number of raw reads and the percentage of microbial, bacterial, viral, fungal, and protozoan reads and then visualised by the Sankey diagrams.

The difference between measured variables (number of raw reads, microbial, bacterial, viral, fungal, and protozoan reads) obtained by nitrocellulose filters and collection liquids was determined using the Wilcoxon signed rank test adjusted by the Bonferroni correction. P-values of less than 0.05 were considered statistically significant. Statistical analyses were performed using IBM^®^ SPSS^®^ for Windows version 26 (SPSS Inc., IBM Company, Chicago, IL, USA) and Excel^®^ for Windows^®^ version 2016 (Microsoft™, Redmond, WA, USA).

## 3. Results

### 3.1. Microbial Identification with MALDI-TOF MS and OmniLog ID System

From the air samples collected on 10 January 2023 with both air sampler types, we cultured 85 bacterial and 36 fungal morphologically distinct isolates (Table 1 and Table 2). All 121 isolates were subjected to the MALDI-TOF MS identification procedure. Due to growth problems, i.e., unsuccessful purification of mixed cultures, only 84 bacterial and 30 fungal isolates could be later subjected to identification by the OmniLog ID System (Table 1 and Table 2). Using MALDI-TOF MS, we were able to identify 90.6% (77/85) of the bacterial isolates at the genus level (MALDI score ≥ 1.70) and 80.0% (68/85) at the species level (MALDI score ≥ 2.00). Using the OmniLog ID System, we were able to identify 92.9% (78/84) of bacterial isolates to the species level. In total, 48.8% (41/84) of species-level and 70.2% (59/84) of genus-level identifications matched with both methods.

Using MALDI-TOF MS to identify fungi, we were able to identify 72.2% (26/36) of isolates to the genus level (MALDI score ≥ 1.70) and 30.6% (21/36) to the species level (MALDI score ≥ 2.00) (Table 2). With the OmniLog ID System, the rate of successful species identification after 48 h of incubation was 13.3% (4/30); after 72 h of cultivation, additionally 3.3% (1/30) were successfully identified (Table 2). In total, 30.0% (9/30) of species-level identification matched with both methods, while 93.3% (28/30) were a genus-level match.

Bacterial isolates that did not match at the species or genus level were subjected to 16S rRNA gene sequencing (Table 3). Only the pure cultures whose species and in particular genera could be separated by the sequencing of the 16S rRNA gene amplicon [39] were included in the analysis: 21 bacterial isolates were sequenced (25.0%, 21/84). By sequencing, we were able to identify 14.3% (3/21) of the isolates to species level and the rest (85.7%, 18/21) to genus level (Table 3). Of the molecular identification at the species level, 1/3 matched the MAL-DI-TOF MS identification and 0/3 matched the OmniLog ID System (Table 3). If we also include the equally possible molecular identifications, the species-level identifications of MALDI-TOF MS and the OmniLog ID System matched in 38.1% (8/21) and 0.0% (0/21), respectively (Table 3). For genus-level identification, MALDI-TOF MS matched molecular identification in 90.5% (19/21), while the OmniLog ID System provided matching results at the genus level in 28.6% (6/21).

### 3.2. Metagenomic Analysis

To cover a wider diversity of the airborne microbial community of a speleotherapeutic cave, we analysed its metagenome. A total of eighteen samples were analysed: six samples per sampling site—air was collected on nitrocellulose filters and in collection liquid in technical duplicates (Table 4). Using the Illumina platform, we were able to detect 7480 bacterial, 294 viral, and 244 eukaryotic taxa, generating between 117,357 and 27,049,966 raw reads per sample (Table 4). In total, 8.4% to 94.7% of the raw reads were microbial reads, with bacterial reads being the most abundant at up to 94.3%. Eukaryotes made up the smallest proportion of all reads, generally <1.0% (Table 4).

The most represented phyla were *Pseudomonadota* with 6.91 million reads (5.2%), *Cressdnaviricota* with 6.67 million reads (5.0%), and *Actinomycetota* with 4.08 million reads (3.0%), followed by *Bacillota* with 3.49 million reads (2.6%) and *Streptophyta* with 1.19 million reads (0.9%). Among the predominant genera, we found *Sphingobium* with 5.76 million reads (4.3%), *Cutibacterium* with 4.07 million reads (3.0%), and *Staphylococcus* with 3.45 million reads (2.6%), followed by *Pseudomonas* with 1.63 million reads (1.2%) and *Rhodopseudomonas* with 1.22 million reads (0.9%).

The differences between the results obtained from nitrocellulose filters and collection liquids were statistically significant for microbial (*p* = 0.001) and bacterial reads (*p* = 0.028), while the differences in the number of raw reads (*p* = 0.553) and viral (*p* = 0.249), fungal (*p* = 0.600), and protozoan reads (*p* = 0.917) were not statistically significant.

#### 3.2.1. Metagenomic Analysis—Nitrocellulose Filters

**Figure 2:**
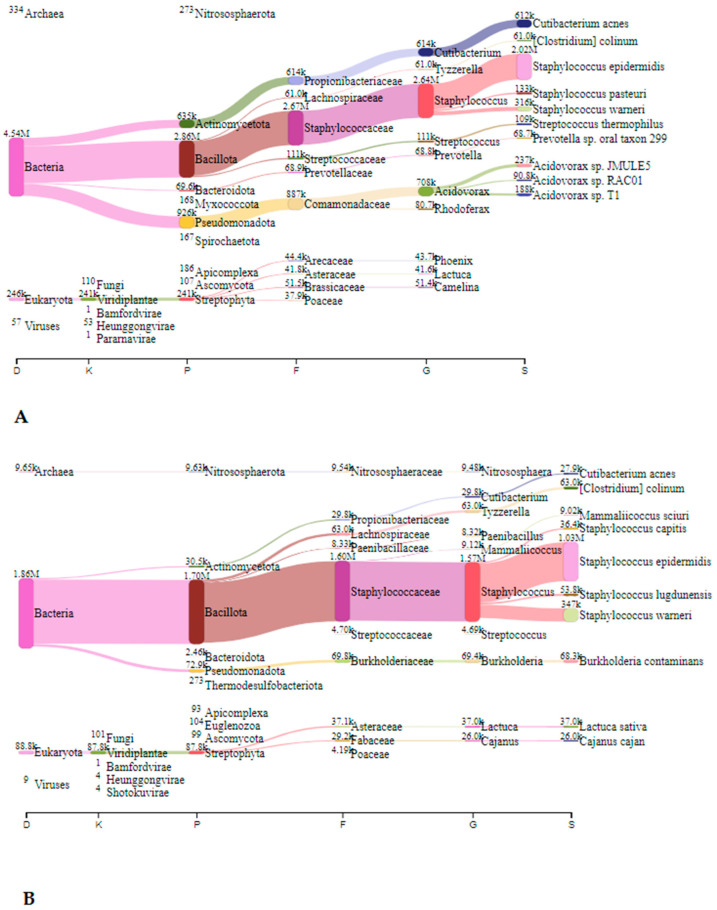
The Sankey visualisation of species present in the Jedilnica sampling site before (**A**) and after (**B**) speleotherapeutic activities using nitrocellulose filters. In both samples, *S. epidermidis* is the most prevalent species. D—domain; K—kingdom; P—phylum; F—family; G—genus; S—species.

In Jedilnica, before speleotherapeutic activities, the most frequently detected species belonged to *Staphylococcus* spp., *Acidovorax* spp., and *Cutibacterium* spp. with 2.64 million (44.2%), 0.71 million (11.9%), and 0.61 million reads (10.2%), respectively (Figure 2A). After the speleotherapeutic activities, *Staphylococcus* spp. remained the most abundant genus with 1.57 million reads (67.6%), followed by *Burkholderia* spp. with 69.4 thousand reads (3.0%) (Figure 2B).

**Figure 3:**
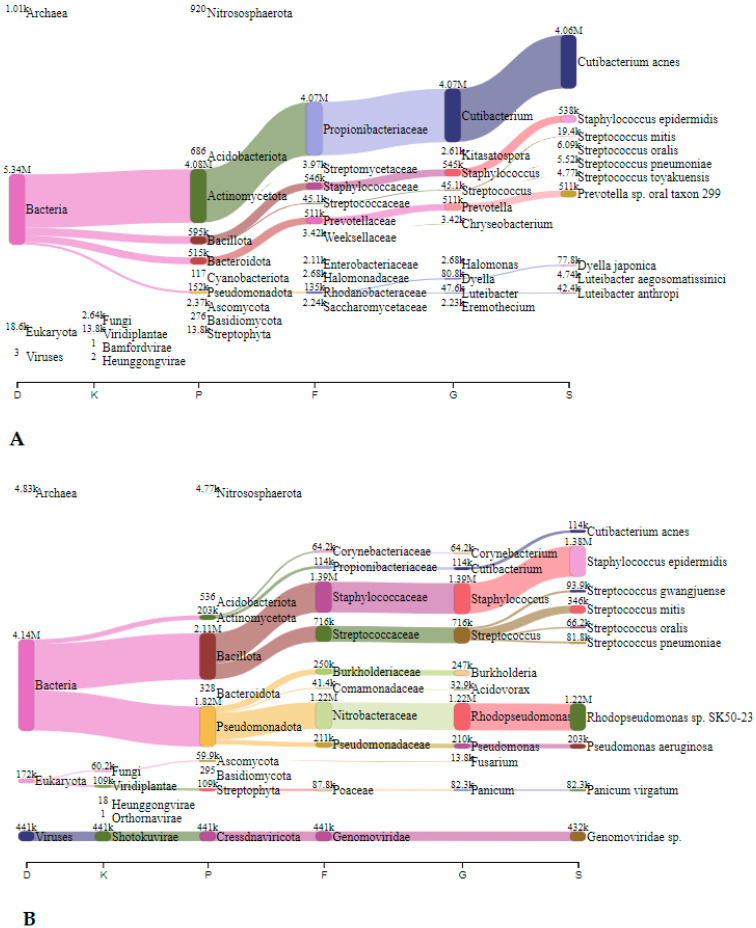
The Sankey visualisation of species present in the Spalnica sampling site before (**A**) and after (**B**) speleotherapeutic activities using nitrocellulose filters. D—domain; K—kingdom; P—phylum; F—family; G—genus; S—species.

In Spalnica, before speleotherapeutic activities, *Cutibacterium* spp. were the most abundant with 4.1 million reads (72.4%), followed by *Staphylococcus* spp. and *Prevotella* spp. with 0.55 million (9.7%) and 0.51 million reads (9.0%), respectively (Figure 3A). After the speleotherapeutic activities, *Staphylococcus* spp. were the most frequently represented with 1.39 million reads (24.4%), followed by *Rhodopseudomonas* spp. and *Steptococcus* spp. with 1.22 million (21.4%) and 0.72 million reads (12.6%), respectively (Figure 3B). They were followed by *Burkholderia* spp. and *Pseudomonas* spp. with 0.25 (4.4%) and 0.21 million reads (3.7%), respectively. Viruses accounted for 7.7%, with the *Genomoviridae* being the most frequently represented with 0.44 million reads (Figure 3B).

**Figure 4:**
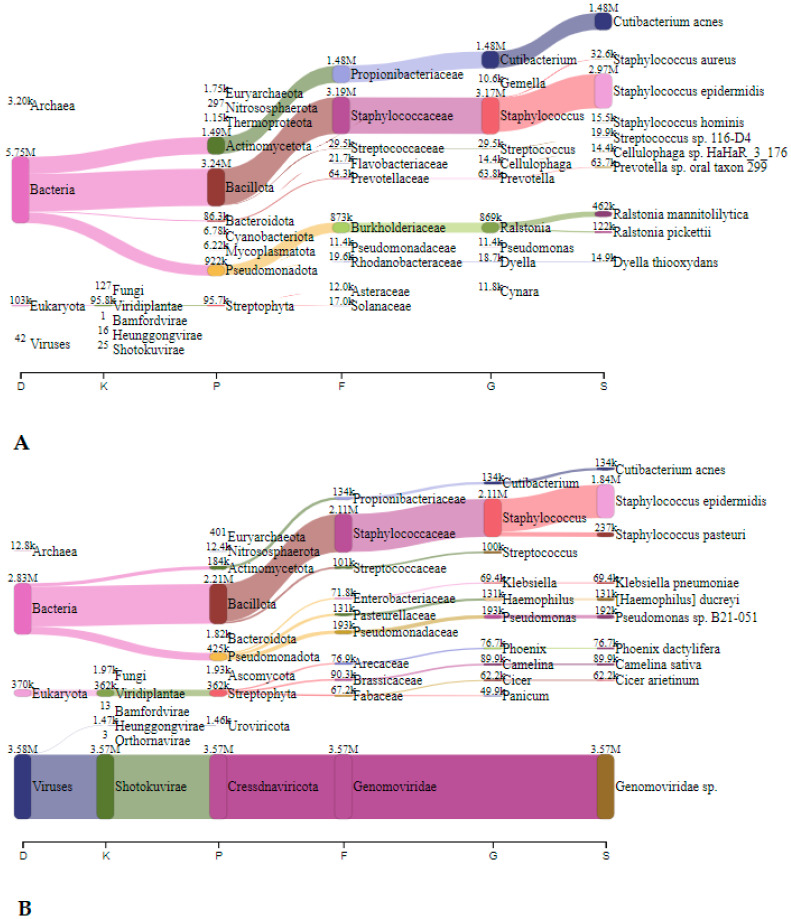
The Sankey visualisation of species present in the Telovadnica sampling site before (**A**) and after (**B**) speleotherapeutic activities using nitrocellulose filters. In both samples, *S. epidermidis* is the most abundant bacterial species. D—domain; K—kingdom; P—phylum; F—family; G—genus; S—species.

In Telovadnica, *Staphylococcus* spp., *Cutibacterium* spp., and *Ralstonia* spp. predominated before speleotherapeutic activities with 3.17 million (43.4%), 1.48 (20.3%), and 0.87 million reads (11.9%), respectively (Figure 4A). After the speleotherapeutic activities, *Staphylococcus* spp. and *Pseudomonas* spp. were the most common, with 1.39 (15.5%) and 0.19 million reads (2.1%), respectively (Figure 4B). Viruses accounted for 39.9%, with the *Genomoviridae* family being the most frequently represented with 3.57 million reads (Figure 4B).

#### 3.2.2. Metagenomic Analysis—Collection Liquid

**Figure 5:**
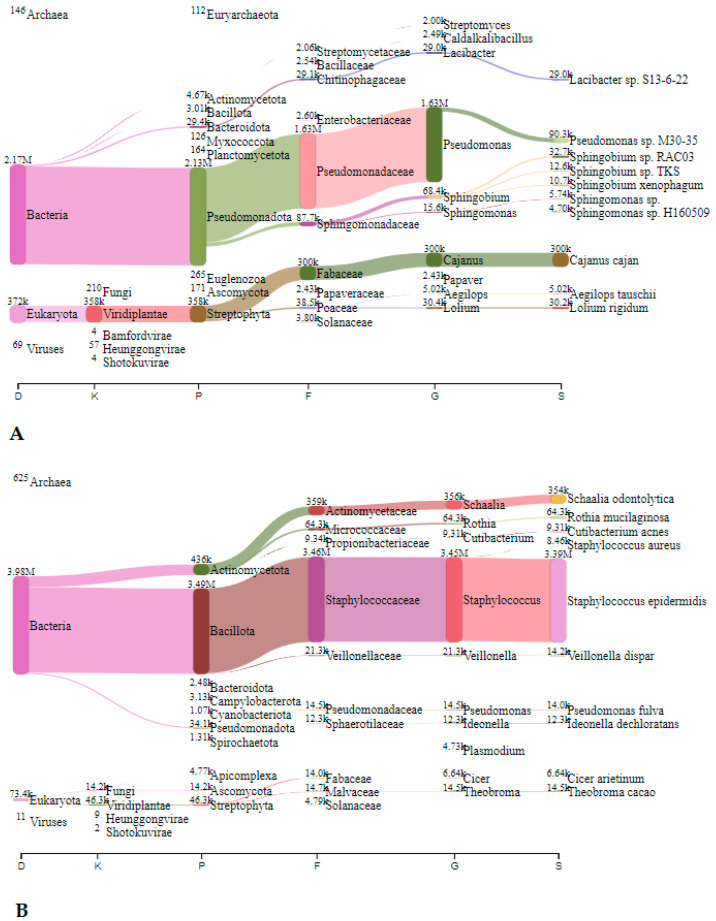
The Sankey visualisation of species present in the Jedilnica sampling site before speleotherapeutic activities using collection liquid in duplicates (**A**,**B**). D—domain; K—kingdom; P—phylum; F—family; G—genus; S—species.

**Figure 6:**
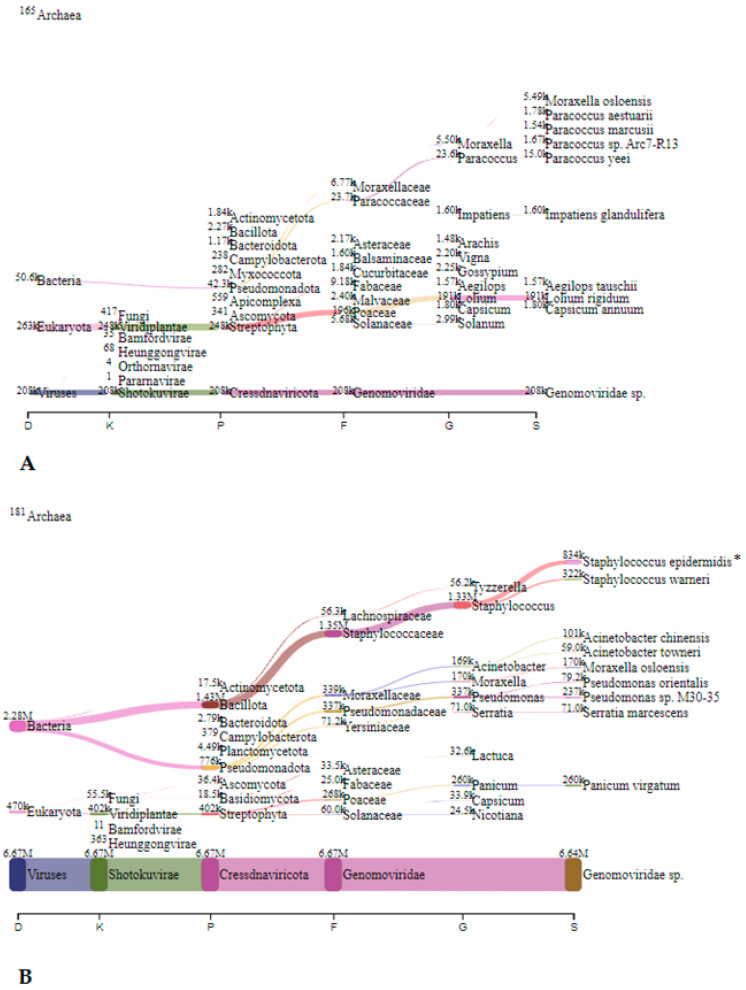
The Sankey visualisation of species present in the Jedilnica sampling site after speleotherapeutic activities using collection liquid in duplicates (**A**,**B**). D—domain; K—kingdom; P—phylum; F—family; G—genus; S—species. * Same species also present in the “before speleotherapeutic activities” sample (Figure 5).

In Jedilnica, before speleotherapeutic activities, *Pseudomonas* was the most frequently detected genus with 1.63 million reads (23.5%), followed by *Cajanus*, plants from the *Fabaceae* family, with 0.3 million reads (4.3%) (Figure 5A). In the duplicated sample, *S. epidermidis* was most abundant with 3.39 million reads (72.6%) (Figure 5B). After speleotherapeutic activities, ryegrass (*Lolium rigidum*) predominated with 0.19 million reads (3.0%) (Figure 6A), while in the duplicated sample, *Genomoviridae* represented 50.7% with 6.64 million reads (Figure 6B).

**Figure 7:**
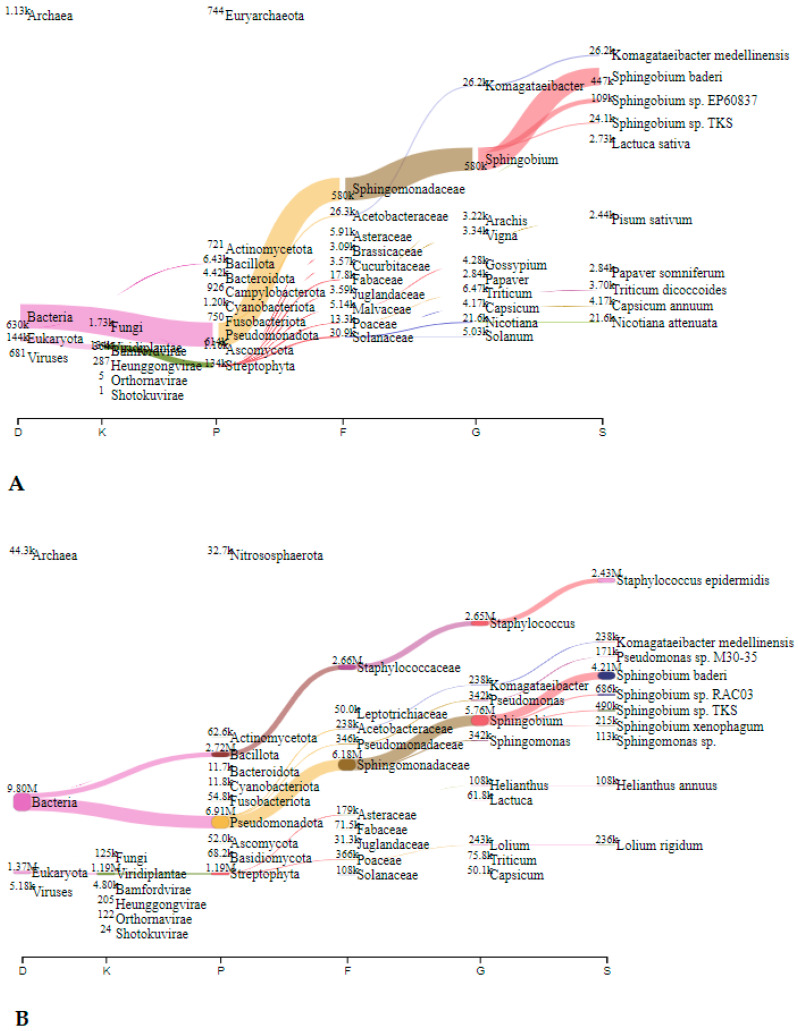
The Sankey visualisation of species present in the Spalnica sampling site before speleotherapeutic activities using collection liquid in duplicates (**A**,**B**). D—domain; K—kingdom; P—phylum; F—family; G—genus; S—species.

**Figure 8:**
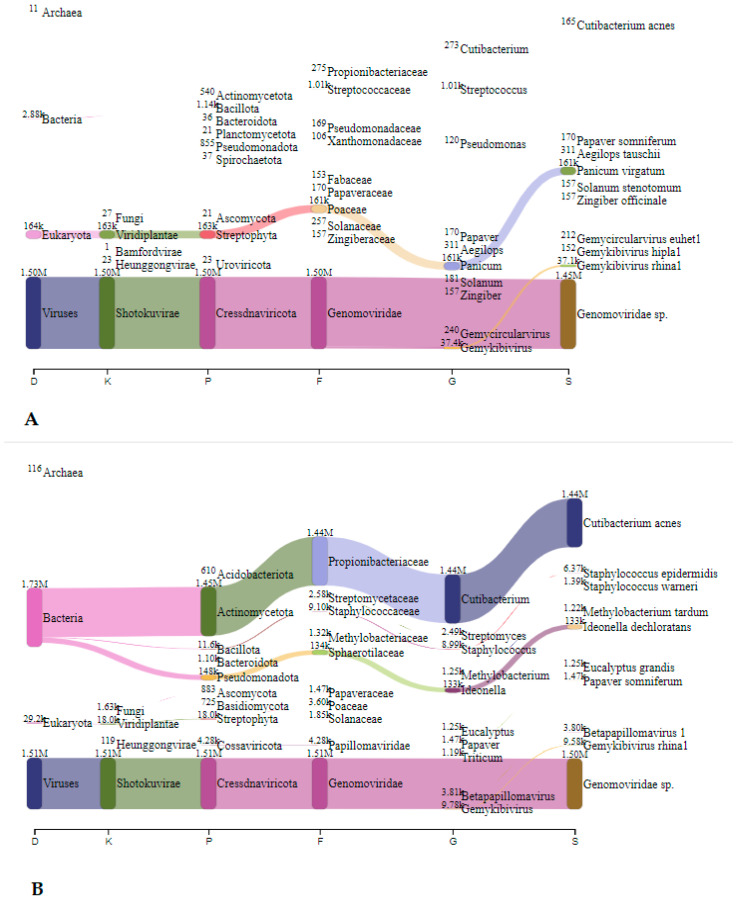
The Sankey visualisation of species present in the Spalnica sampling site after speleotherapeutic activities using collection liquid in duplicates (**A**,**B**). D—domain; K—kingdom; P—phylum; F—family; G—genus; S—species.

In Spalnica, before speleotherapeutic activities, *Sphingobium* was the most frequently detected genus with 0.58 million reads (12.9%), followed by *Kromagataeibacter medellinensiss* with 26.2 thousand reads (0.6%) (Figure 7A). In the duplicated sample, *Sphingobium* was also the most abundant with 5.76 million reads (21.3%), followed by *S. epidermidis* with 2.43 million reads (9.0%) (Figure 7B). After speleotherapeutic activities, *Genomoviridae* predominated with 1.45 million reads (44.3%), followed by switchgrass (*Pancium virgatum*) with 0.16 million reads (4.9%), while in the duplicated sample, *Genomoviridae* was still the most abundant with 1.50 million reads (2.5%), followed by *C. acnes* with 1.44 million reads (2.4%) (Figure 8B).

**Figure 9:**
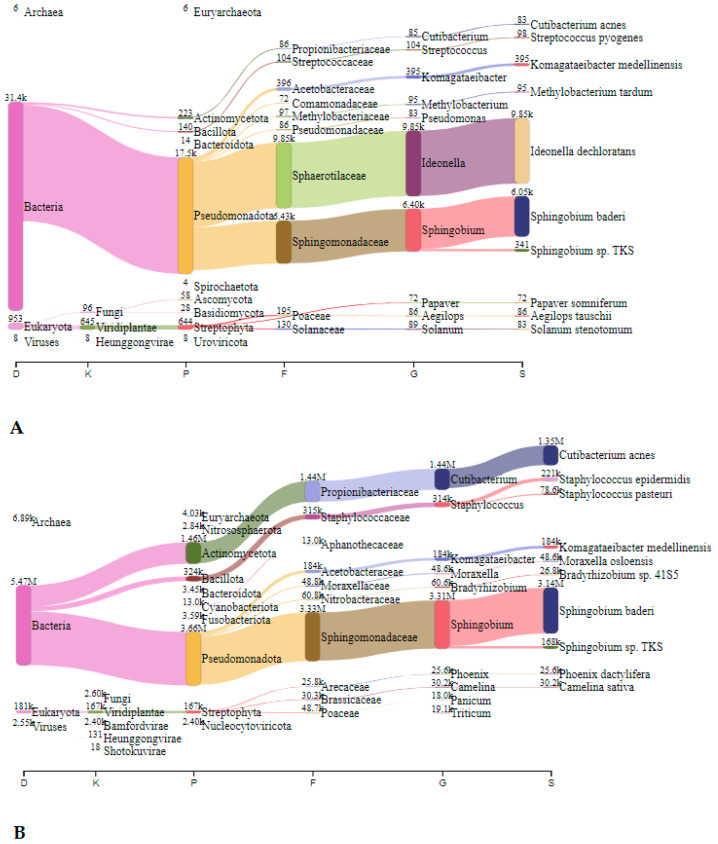
The Sankey visualisation of species present in the Telovadnica sampling site before speleotherapeutic activities using collection liquid in duplicates (**A**,**B**). D—domain; K—kingdom; P—phylum; F—family; G—genus; S—species.

**Figure 10:**
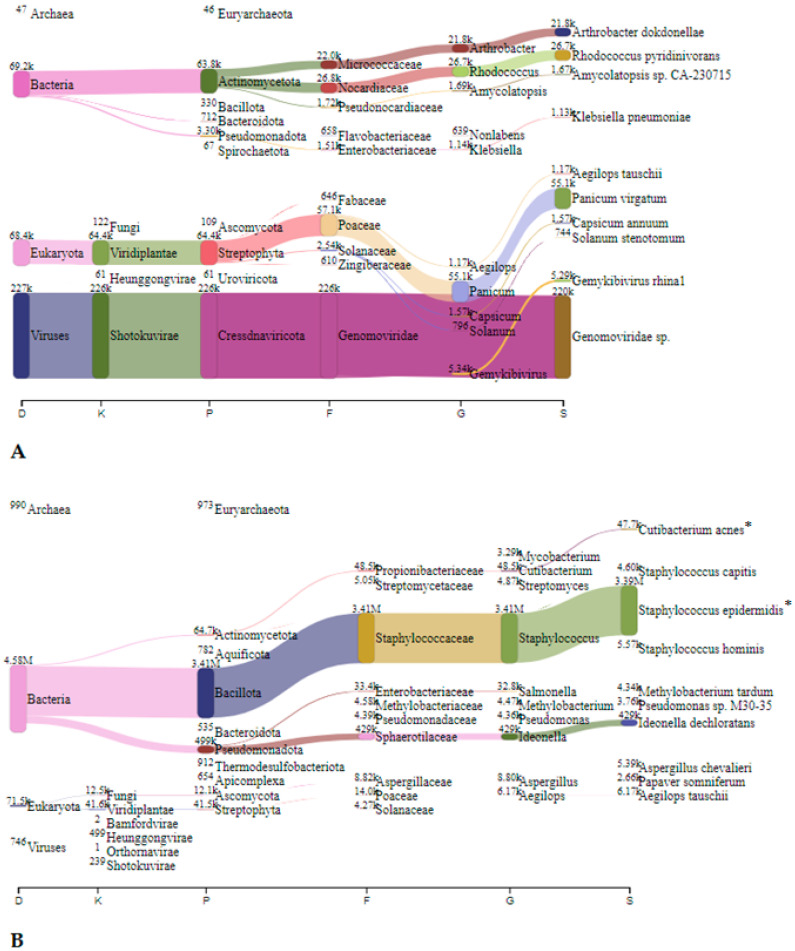
The Sankey visualisation of species present in the Telovadnica sampling site after speleotherapeutic activities using collection liquid in duplicates (**A**,**B**). D—domain; K—kingdom; P—phylum; F—family; G—genus; S—species. * Same species also present in the “before speleotherapeutic activities” sample (Figure 9).

In Telovadnica, before speleotherapeutic activities, *Ideonella dechloratans* was the most frequently detected species with 9.85 thousand reads (8.4%), followed by *Sphingobium baderi* with 6.05 thousand reads (5.2%) (Figure 9A). In the duplicated sample, *Sphingobium baderi* was also the most abundant with 3.14 million reads (39.2%), followed by *C. acnes* with 1.35 million reads (16.8%) (Figure 9B). After speleotherapeutic activities, viruses predominated with 0.227 million reads, representing 2.5% (Table 4, Figure 10A). Prokaryotes and Eukaryotes were equally represented with 69.3 thousand (0.8%) and 68.4 thousand (0.8%) reads, respectively (Figure 10A). In the duplicated sample, viruses were represented with only 746 reads (0.008%), while bacteria dominated with *S. epidermidis* being the most abundant with 3.39 million reads (37.9%) (Figure 10B).

## 4. Discussion

### 4.1. Identification of Bacteria by MALDI-TOF MS and OmniLog ID System

With MALDI-TOF MS, we were able to identify 80.0% of bacterial isolates to species level; with the OmniLog ID System, we were able to identify even 12.9 percentage points more (92.9%), which indicates a greater success rate of the OmniLog ID System. However, despite the high proportion of bacteria identified with both methods, the identifications to the species level matched in less than half of the cases (48.8%). For these mismatched and subsequently sequenced (16S rRNA) isolates, MALDI-TOF MS identification was proven successful to species level in 38.1% of cases, while the OmniLog ID System did not provide a single correct identification to species level (0/21). This implies that MALDI-TOF MS provides a more reliable identification, regardless of the origin of the isolate (clinical or environmental isolates). Our results showed that MALDI-TOF MS is a more robust identification method when it comes to species-level identification, while the OmniLog ID System is acceptable for genus-level identification which, in our case, matched MALDI-TOF MS in 70.2%. The ability of the OmniLog ID System to correctly identify bacterial genera has already been described in other studies [40,41]. 

MALDI-TOF MS is suitable for the identification of microorganisms associated with humans, e.g., staphylococci, as previously described in other studies [5,42]. This is particularly important for microbiological air monitoring in speleotherapeutic caves, as human-associated and medically important microorganisms are targeted [10,31,43,44]. A good example is the misidentification of a strain of *S. warneri* as *S. aureus* (Isolate 80) by the OmniLog ID System. *S. aureus* is one of the most important pathogenic bacteria and has many clinical and epidemiological consequences. Among other things, it plays the role of an indicator organism in the microbiological monitoring of the hospital environment, indicating inadequate conditions for the performance of certain medical activities [2,45,46,47]. The detection of *S. aureus* in a speleotherapeutic cave could result in limited access for therapeutic purposes and the search for and sanitisation of the source. MALDI-TOF MS and 16S rRNA gene amplicon sequencing identified the abovementioned isolate as *S. warneri*, which as a commensal part of the skin microbiota does not pose a risk of colonisation and/or infection to patients [48,49]. In highly anthropised caves, we expect to find staphylococci and other members of the human core microbiota [5,6,10]. 

MALDI-TOF MS is a better choice for the identification of bacterial isolates than the OmniLog ID System, as correct identification at the genus level is not sufficient in a speleotherapeutic cave, which is actually a special type of hospital environment. Similar results were noted by Sandle et al. [41] where the OmniLog ID System failed in the identification of *Micrococcaceae*, which are important in clinical and pharmaceutical environments. In our study, most of the bacterial genera misidentified by the OmniLog ID System were from the order *Micrococcales* and were already misidentified at the family level. One example is the genus *Micrococcus*, which was misidentified as *Janibacter*, *Microbacterium*, *Brevibacterium*, and *Bacillus*. All these genera, except *Bacillus*, belong to different families of *Micrococcales*. Our study and the study by Sandle et al. [41] suggest that the identification of *Staphylococcus*, *Micrococcus*, and related genera should not be based solely on the OmniLog ID System. In most studies, the OmniLog ID System was used very successfully for the physiological characterisation of specific isolates that had previously been identified by another method, or the identification by the OmniLog ID System was additionally verified by sequencing the 16S rRNA gene [13,50,51]. 

In speleotherapeutic caves, we would recommend MALDI-TOF-MS for routine identification and the OmniLog ID System only if identification to genus level is considered sufficient. Otherwise, identification to species level should be verified by another method, possibly by sequencing the 16S rRNA gene. 

### 4.2. Identification of Fungi by MALDI-TOF MS and OmniLog ID System

Using only MALDI-TOF MS to identify fungi, we were able to identify 72.2% of isolates at the genus level (MALDI score ≥ 1.70) and 30.6% at the species level (MALDI score ≥ 2.00). We would likely achieve a higher percentage of mould identifications if we created our own mass spectra library with a broader genus/species database; studies showed that using a user-developed library instead of the manufacturer’s commercially available library significantly increases identification success, as more than 95% of fungal isolates can be identified [52,53]. 

With the OmniLog ID System, the species identification rate of 16.7% was significantly lower than that with MALDI-TOF MS. Nevertheless, 30.0% of identifications matched at the species level with both methods, while 93.3% matched at the genus level, indicating comparable success in genus-level identification. However, the results obtained with the OmniLog FF MicroPlate must also be further verified morphologically, which is facilitated by the mycological atlas integrated into the software, but the method still requires considerable knowledge of classical mycology. The only results in which the genera did not match were obtained with the YT MicroPlate in the identification of basidiomycetous yeasts: Fungal isolates 8 and 9 were both identified by MALDI-TOF MS as *Cutaneotrichosporon dermatis*, while OmniLog’s YT MicroPlate identified them as *Hannaella luteola* and *Bullera alba*, respectively. This identification is already a mismatch at the order level, as *Cutaneotrichosporon* belongs to the *Trichosporonales*, while *Hannaella* and *Bullera* both belong to the *Tremellales*. However, since only two isolates are involved here, a larger collection of basidiomycetous yeast isolates should be tested to verify the significance of this discrepancy. As with bacterial identification, fungal identification with the OmniLog ID System should be accompanied by another identification method, at least morphology, to achieve better accuracy. Superior to MALDI-TOF MS, the OmniLog ID System provides additional metabolic information that can be used for ecological or biotechnological studies and applications [54,55,56]. 

As far as the identification of microorganisms is concerned, MALDI-TOF MS or the OmniLog ID System alone is not an ideal identification approach, especially in environmental microbiology [7,8,40,57]. To accurately identify microorganisms at the species level, researchers today increasingly use a combination of standard culture-based and visual observation methods with genetic techniques that allow differentiation between species and strains of microorganisms at the molecular level [11,22]. A number of methods have been proposed for the optimal identification of microorganisms, each with its own advantages and limitations [11]. In addition to MALDI-TOF MS, the OmniLog ID System, and genome-based methods, other successful methods range from morphology and gas–liquid chromatography of cellular fatty acids to the use of optical methods for the label-free detection of bacteria [3,58,59,60]. Overall, however, these studies emphasise the need for a multi-method approach to microbial identification that combines the strengths of individual methods to achieve optimal results. In addition to species identification, resistance profiles and phylogenetic comparisons could provide more precise information on the origin of the isolated microorganisms.

### 4.3. Metagenomic Analysis and Anthropogenic Indicators

We detected 100 times more bacterial species with the metagenomic approach than with the culture-based approach (7480 vs. 74 species), which is consistent with other studies [61,62], proving that the metagenomic approach reveals greater species diversity than the culture-based approach. As in other metagenomics-based studies of caves [33,34,35,57,63,64], *Pseudomonadota*, *Actinomycetota*, and *Bacillota* were the most abundant bacterial phyla, but interestingly in our case, the viral phylum *Cressdnaviricota* was the second most abundant phylum, just after *Pseudomonadota*. These small, circular, single-stranded DNA viruses have also previously been found in caves, in association with bats [65,66,67]. *Genomoviridae*, a family and species of cressdnaviruses, was found in the Sežana Hospital Cave mainly in Spalnica and Telovadnica after speleotherapeutic activities and accounted for up to 50.7% of the reads. One possible explanation for their occurrence is that these viruses are not only associated with bats but also with fungi [68,69], which accounted for up to 14.5% of the cultivable microorganisms at these sampling sites [10]—the fungal spores were probably aerosolised together with the viruses during speleotherapeutic activities. 

The abundant presence of staphylococci revealed by culture-dependent methods, especially *S. epidermidis* and *S. warneri* [10], was confirmed by metagenomic analysis. Metagenomics has helped us to uncover the slow-growing anthropogenic indicators, such as *Cutibacterium acnes*, which is part of the human skin microbiota and otherwise requires special conditions for its cultivation [48]. *C. acnes* was detected in all air samples collected on nitrocellulose filters but interestingly was present in higher numbers in the “before patient” samples. The clearest example is the sampling site in Spalnica, where we detected 4.06 million reads (72.4%) specific for *C. acnes* before the speleotherapeutic activities, while the number of reads fell to 0.114 million (2.0%) afterwards. The reason for this is not entirely clear, but it shows that the Sežana Hospital Cave has a highly anthropised aerobiome background, as was also shown in a previous culture-based study [10]. 

Among the cultivable microorganisms, metagenomic analysis detected the *Streptococcus mitis* group and *Moraxella osloensis*—potential anthropogenic indicators previously cultured from cave air samples in *Postojnska jama* [5]. This time, the *S. mitis* group and *M. osloensis* could not be detected by cultivation, which speaks in favour of the advantages of the integrated approach [70]. Among the slow-growing bacteria that were also not detected by the culture-based method were *Burkolderia* spp. which were detected in Jedilnica and Spalnica up to 4.4% after speleotherapeutic activities. *Burkolderia* spp. can be associated with humans but have been found in both pristine and anthropised caves [71,72,73], so we cannot identify these bacteria as an indicator of cave anthropisation.

Metagenomic analysis has shown that microorganisms associated with humans are present and widespread in the Sežana Hospital Cave regardless of human presence, just as culture-based methods have shown us: culture- and metagenomics-based methods complement each other and show that the aerobiota in the Sežana Hospital Cave is based on human-associated microbial species [10].

### 4.4. Sample Types and Reproducibility

Metagenomics-based approaches enable a comprehensive understanding of microbial diversity in caves [74]. However, the taxonomic distribution in different caves can be difficult to compare due to differences in DNA extraction protocols, sequencing technologies, and bioinformatics tools [74,75]. On the other hand, culture-based approaches can provide a more realistic representation of species diversity when used in combination with metagenomic techniques [70]. This integrated approach has already proven successful in the study of microbial diversity and function in cave environments [70] and in this study in relation to anthropogenic indicators. The lack of standardisation of the entire metagenomic process makes interpretation difficult [75,76], especially when dealing with a small number of reads per sample or per specific taxonomic unit. Hillmann et al. [77] suggested at least 0.5 million reads as the limit for the minimum information content for a successful taxonomic mapping, while Jo et al. [23] suggested a number ten times higher, i.e., 5 million reads. Using the Hillmann criterion [77], we were able to taxonomically map all samples except “Telovadnica—before patients” from collection liquid, where we recorded only 117,357 reads. Using the Jo criterion [23], a sufficient number of reads was achieved in 72.2% of the samples. Of the information-insufficient samples (27.8%), only one sample is a nitrocellulose filter; the rest are collection liquids, indicating a better suitability of nitrocellulose filters for metagenomic air analysis, as they provide more raw reads. The filters also proved to be more suitable in terms of the percentage of microbial reads, as this difference was also statistically significant (*p* = 0.001); with nitrocellulose filters, we obtained between 76% and 94.7% of microbial reads, while with collection liquid, we obtained between 4.1% and 86.9% of microbial reads. Overall, we obtained an average of 83.2% microbial reads with nitrocellulose filters, which is 33.6 percentage points higher than with collection liquid. Based on our results for metagenomic air analysis, we recommend collecting air samples with nitrocellulose filters, as this approach yields more high-quality reads. 

The differences between the individual replicates are relatively large and, in our opinion, indicate the poor suitability of saline solution as a carrier for air sampling for metagenomic bacteriological studies in caves. Another reason could be the heterogeneity of the air and the resulting poor reproducibility. The predominant microorganisms differ in most cases between replicates, even at the phylum level. For example, in the case of Jedilnica sampled before speleotherapeutic activities, one sample is dominated by *Pseudomonadota* with 2.13 million reads, while the duplicate is dominated by *Bacillota* with 3.49 million reads, with only 34.1 thousand reads for *Pseudomonadota*. The situation is similar with Spalnica sampled before speleotherapeutic activities: *Actinomycetota* dominates with 1.45 million reads, while it is only represented with 540 reads in a duplicated sample. Better reproducibility from the collection liquid was achieved for viruses, which were always present in both replicates. To obtain a more reliable pool of results and draw more solid conclusions about the reproducibility and suitability of nitrocellulose filters and collection liquids for metagenomic analyses of cave air, we would need to test a larger number of samples, but nevertheless, based on our results, we would recommend the use of nitrocellulose filters instead of collection liquids as a step towards the standardisation of methods in cave aerobiology.

### 4.5. Limitations

Like all studies, this study has certain limitations. A larger number of air samples would provide a more reliable pool of results to draw more solid conclusions about the combination of culture-based and metagenomic approaches in analysing microbial populations in speleotherapeutic caves. A larger number of isolates—perhaps from multiple samplings—identified using MALDI-TOF MS and the OmniLog ID System could also provide more reliable conclusions about the success of identification and the appropriateness of the two methods in the biocontamination control of speleotherapeutic caves. Extending our research to non-anthropised caves would also provide greater insight into the structure and dynamics of cave aerobiota independent of humans.

## 5. Conclusions

Our results show that MAL-DI-TOF MS is a reliable tool for the identification of microorganisms in speleotherapeutic caves, as its comprehensive database contains mainly human-associated and clinically important microbial species. The use of the OmniLog ID System is recommended only in combination with other identification methods, such as 16S rRNA gene amplicon sequencing, which can be used to verify identification to the species level. The metagenomic approach in the analysis of aerobiota produced similar results to the culture-based methods in the assessment of cave anthropisation, suggesting that a combination of strategies is optimal as one method complements the other. Apart from *Staphylococcus* spp., we were able to detect the non-cultivable part of aerobiota associated with humans, in particular *Cutibacterium acnes*. Metagenomic analysis also revealed the presence of cressdnaviruses for the first time in the air of Sežana Hospital Cave. Our results show that the collection of air samples using nitrocellulose filters provides better results in terms of the number of classified microbial reads than the use of collection liquids.

## Figures and Tables

**Table 1:** Identification of bacterial isolates with MALDI-TOF MS and the OmniLog ID System. Discrepant identification at the genus level is marked in grey.

	MALDI-TOF MS		OmniLog ID System		
Isolate ID	Identification	Score *	Identification	Probability ^§^ (%)	Similarity Index
1	*Acinetobacter guillouiae*	2.44	*Acinetobacter guillouiae*	65.5	0.520
2	*Acinetobacter guillouiae*	2.26	*Acinetobacter guillouiae*	76.6	0.556
3	*Acinetobacter johnsonii*	2.43	*Advenella incenata*	58.5	0.524
4	*Acinetobacter lwoffii*	2.27	*Acinetobacter lwoffii*	81.2	0.834
5	*Advenella kashmirensis*	1.88	*Staphylococcus epidermidis*	73.4	0.517
6	*Aerococcus viridans*	2.23	*Streptococcus orisratti*	89.9	0.679
7	*Bacillus mycoides*	1.95	*Paenibacillus anaericanus*	70.4	0.704
8	*Bacillus mycoides*	1.89	*Bacillus pseudomycoides/cereus*	80.6	0.549
9	*Bacillus mycoides*	2.40	*Bacillus pseudomycoides/cereus*	65.1	0.522
10	*Bacillus pumilus*	2.04	*Bacillus safensis/pumilus*	92.6	0.701
11	*Enterococcus moraviensis*	1.83	*Enterococcus gallinarum*	85.2	0.674
12	*Exiguobacterium* sp.	2.19	*Bacillus halodurans*	95.2	0.589
13	*Filifactor villosus*	1.39	*Williemsia muralis*	84.9	0.787
14	*Kocuria rhizophila*	2.11	*Rhodococcus fasclans*	88.8	0.734
15	*Kocuria rosea*	1.39	*Staphylococcus capitis*	78.9	0.585
16	*Microbacterium maritypicum*	1.61	*Microbacterium liquefaciens*	61.0	0.610
17	*Microbacterium oxydans*	2.39	*Microbacterium maritypicum*	94.8	0.582
18	*Microbacterium phyllosphaerae*	2.01	*Microbacterium maritypicum*	82.9	0.518
19	*Microbacterium phyllosphaerae*	1.93	*Microbacterium marytipicum*	88.7	0.509
20	*Microbacterium phyllosphaerae*	1.86	*Microbacterium marytipicum*	95.0	0.596
21	*Micrococcus flavus*	2.35	*Dietzia maris*	75.1	0.506
22	*Micrococcus flavus*	2.32	*Nesterenkonia sandarakina*	89.8	0.787
23	*Micrococcus luteus*	2.39	*Micrococcus yunnanensis*	96.9	0.808
24	*Micrococcus luteus*	2.27	*Micrococcus yunnanensis*	70.4	0.704
25	*Micrococcus luteus*	2.09	*Micrococcus luteus*	87.0	0.542
26	*Micrococcus luteus*	2.24	*Micrococcus luteus*	N	0.193
27	*Micrococcus luteus*	2.22	*Kytococcus schroeteri*	77.2	0.772
28	*Micrococcus luteus*	2.25	*Micrococcus luteus*	51.8	0.659
29	*Micrococcus luteus*	2.27	*Micrococcus luteus*	N	0.454
30	*Micrococcus luteus*	2.30	*Micrococcus luteus*	N	0.275
31	*Micrococcus luteus*	2.23	*Micrococcus luteus*	94.4	0.631
32	*Micrococcus luteus*	2.32	*Micrococcus luteus*	69.9	0.566
33	*Micrococcus luteus*	2.27	*Microbacterium marytipicum*	82.9	0.518
34	*Micrococcus luteus*	2.14	*Micrococcus luteus*	90.8	0.638
35	*Micrococcus luteus*	2.40	*Brevibacterium otitidis*	74.1	0.741
36	*Micrococcus luteus*	2.38	*Micrococcus yunnanensis*	74.1	0.741
37	*Micrococcus luteus*	2.27	*Virgibacillus salexigens*	N	0.271
38	*Micrococcus luteus*	2.02	*Brevundimonas otitidis*	74.9	0.749
39	*Micrococcus luteus*	2.2	*Bacillus krulwichiae*	NA	0.235
40	*Micrococcus luteus*	2.12	*Janibacter anophelis/hoylei*	85.3	0.803
41	*Micrococcus* sp.	2.10	*Micrococcus luteus*	75.3	0.502
42	*Pantoea eucrina*	2.10	*Pantoea dispersa*	91.3	0.250
43	*Peribacillus simplex*	2.04	*Bacillus simplex/butanolivorans*	50.9	0.509
44	*Pseudarthrobacter oxydans*	2.39	*Janibacter anophelis/hoylei*	87.8	0.613
45	*Pseudoclavibacter helvolus*	1.97	*Virgibacillus salexigens*	86.5	0.734
46	*Pseudomonas fluorescens*	1.97	*Pseudomonas marginalis*	84.6	0.54
47	*Pseudomonas gessardi*	1.79	*Pseudomonas fluorescens*	75.3	0.519
48	*Pseudomonas putida*	1.42	*Brachybacterium muris*	87.5	0.617
49	*Pseudomonas putida*	1.45	*Serpens flexibilis*	86.8	0.688
50	*Pseudomonas trivialis*	1.37	*Brachybacterium muris*	87.1	0.529
51	*Rahnella aquatilis*	2.09	*Rahnella aquatilis*	81.7	0.516
52	*Serratia proteamaculans*	2.23	*Serratia proteamaculans*	84.1	0.507
53	*Sphingomonas faeni*	1.51	*Staphylococcus epidermidis*	76.2	0.548
54	*Staphylococcus capitis*	2.25	*Staphylococcus capitis*	75.1	0.512
55	*Staphylococcus capitis*	2.30	*Staphylococcus capitis*	79.8	0.529
56	*Staphylococcus capitis*	2.18	*Staphylococcus capitis*	79.6	0.608
57	*Staphylococcus capitis*	2.16	*Staphylococcus capitis*	70.7	0.503
58	*Staphylococcus capitis*	2.14	*Staphylococcus capitis*	72.8	0.501
59	*Staphylococcus capitis*	2.16	*Staphylococcus capitis*	89.9	0.613
60	*Staphylococcus capitis*	2.15	*Staphylococcus capitis*	92.0	0.689
61	*Staphylococcus chonii*	2.25	*Staphylococcus saprophyticus*	89.3	0.529
62	*Staphylococcus epidermidis*	2.09	*Staphylococcus epidermidis*	69.3	0.508
63	*Staphylococcus epidermidis*	2.09	*Staphylococcus epidermidis*	87.5	0.642
64	*Staphylococcus epidermidis*	2.14	*Staphylococcus epidermidis*	73.4	0.517
65	*Staphylococcus epidermidis*	2.10	*Staphylococcus epidermidis*	84.9	0.591
66	*Staphylococcus hominis*	2.17	*Staphylococcus hominis*	81.9	0.523
67	*Staphylococcus hominis*	2.16	*Staohylococcus hominis*	78.8	0.543
68	*Staphylococcus hominis*	2.27	*Paenibacillus provencensis*	87.0	0.704
69	*Staphylococcus hominis*	2.30	*Staphylococcus hominis*	73.5	0.523
70	*Staphylococcus hominis*	2.25	*Staphylococcus hominis*	74.1	0.522
71	*Staphylococcus hominis*	2.10	*Staphylococcus hominis*	75.1	0.512
72	*Staphylococcus hominis*	2.22	*Staphylococcus hominis*	69.6	0.500
73	*Staphylococcus hominis*	2.38	*Staphylococcus hominis*	89.7	0.549
74	*Staphylococcus hominis*	2.34	*Staphylococcus hominis*	72.8	0.518
75	*Staphylococcus hominis*	2.13	*Staphylococcus hominis*	88.7	0.522
76	*Staphylococcus pettenkoferi*	2.25	*Staphylococcus capitis*	88.3	0.529
77	*Staphylococcus schleiferi*	2.20	*Staphylococcus schleiferi* sp. *coagulans*	99.4	0.821
78	*Staphylococcus warneri*	2.06	*Staphylococcus warneri*	74.0	0.510
79	*Staphylococcus warneri*	2.12	*Staphylococcus warneri*	95.0	0.610
80	*Staphylococcus warneri*	2.00	*Staphylococcus aureus*	96.0	0.638
81	*Stenotrophomonas maltophilia*	2.13	*Stenotrophomonas maltophilia*	71.3	0.513
82	*Stenotrophomonas rhizophila*	2.00	*Stenotrophomonas rhizophila*	95.3	0.610
83	*Streptomyces anulatus*	2.09	*Rahnella aquatilis*	81.4	0.514
84	*Streptomyces badius*	2.08	NA	NA	NA
85	*Streptomyces violaceoruber*	1.38	*Paenibacillus sanguinis*	88.1	0.674

NA—not applicable (subcultivations not successful). N—identification probability not available. * A MALDI score of >2.0 indicates a reliable identification at the species level; a MALDI score between 1.70 and 1.99 indicates a reliable identification at the genus level. ^§^ A 50.0% probability after 22 h of incubation indicates identification to species level.

**Table 2:** Identification of fungal isolates with MALDI-TOF MS and the OmniLog ID System. The identification of the moulds was verified by morphological identification [37,38]. Discrepant identification at the genus level is marked in grey.

	MALDI-TOF MS		OmniLog ID System		
Isolate ID	Identification	Score *	Identification	Incubation Time (h)	Similarity Index ^§^
1	*Aspergillus amstelodami*	1.55	*Aspergillus violaceus*	168	0.258
2	*Aspergillus flavus*	1.85	*Aspergillus flavus*	48	0.583
3	*Aspergillus fumigatus*	2.02	*Aspergillus fumigatus*	48	0.711
4	*Aspergillus fumigatus*	1.89	*Aspergillus fumigatus*	72	0.652
5	*Aspergillus glaucus*	2.20	*Aspergillus rugulosus*	96	0.316
6	*Cladosporium cladosporioides*	1.50	*Cladosporium sphaerospermum*	96	0.285
7	*Cladosporium* sp.	1.71	*Cladosporium tenuissimum*	96	0.566
8	*Cutaneotrichosporon dermatis*	2.20	*Hannaella luteola*	48	0.832
9	*Cutaneotrichosporon dermatis*	2.28	*Bullera alba*	48	0.868
10	*Meyerozyma guilliermondii*	2.18	*Meyerozyma guilliermondii*	48	0.744
11	*Meyerozyma guilliermondii*	2.34	*Meyerozyma guilliermondii*	48	0.699
12	*Penicillium brevicompactum*	1.90	*Penicillium freii*	48	0.53
13	*Penicillium brevicompactum*	2.07	*Penicillium chrysogenum*	168	0.535
14	*Penicillium brevicompactum*	2.24	*Penicillium freii*	48	0.423
15	*Penicillium brevicompactum*	1.95	*Penicillium viridictum*	96	0.575
16	*Penicillium chrysogenum*	1.47	NA	NA	NA
17	*Penicillium chrysogenum*	1.39	NA	NA	NA
18	*Penicillium chrysogenum*	1.68	*Penicillium chrysogenum*	96	0.535
19	*Penicillium chrysogenum*	1.49	NA	NA	NA
20	*Penicillium chrysogenum*	1.48	NA	NA	NA
21	*Penicillium chrysogenum*	1.67	NA	NA	NA
22	*Penicillium chrysogenum*	1.84	*Penicillium thomii*	168	0.425
23	*Penicillium chrysogenum*	1.93	*Penicillium chrysogenum*	48	0.671
24	*Penicillium chrysogenum*	1.78	*Penicillium chrysogenum*	168	0.552
25	*Penicillium chrysogenum*	1.89	*Penicillium freii*	48	0.438
26	*Penicillium chrysogenum*	1.93	NA	NA	NA
27	*Penicillium commune*	2.45	*Penicillium commune*	96	0.526
28	*Penicillium commune*	1.86	*Penicillium cyclopium*	168	0.22
29	*Penicillium digitatum*	1.75	*Penicillium javanicum*	96	0.543
30	*Penicillium digitatum*	2.27	*Penicillium tricolor*	72	0.425
31	*Penicillium italicum*	1.74	*Penicillium freii*	48	0.585
32	*Penicillium italicum*	1.53	*Penicillium hirsutum*	168	0.321
33	*Penicillium italicum*	1.80	*Penicillium commune*	48	0.293
34	*Penicillium italicum*	2.82	*Penicillium hirsutum*	96	0.323
35	*Penicillium* sp.	1.72	*Penicillium citrinum*	168	0.411
36	*Penicillium* sp.	1.48	*Penicillium citrinum*	48	0.430

NA—not applicable (subcultivations not successful). * A MALDI score of >2.0 indicates a reliable identification at the species level; a MALDI score between 1.70 and 1.99 indicates a reliable identification at the genus level. ^§^ A similarity index of ≥0.7 after 48 h, ≥0.65 after 72 h, or ≥0.6 after 96 h of incubation in combination with the morphological characteristics indicates identification to species level.

**Table 3:** Molecular identification of discrepant MALDI-TOF MS and OmniLog ID System identifications related to results of the 16S rRNA gene sequencing. Successful molecular species-level identifications are marked in grey.

	MALDI-TOF MS		OmniLog ID System		16S rRNA Sequencing		
Isolate ID	Identification	Score *	Identification	Probability (%)	Identification	Probability (%) ^§^	Equally Possible Molecular Identifications
6	*Aerococcus viridans*	2.23	*Streptococcus orisratti*	89.9	*Aerococcus* sp.	98.9	*A. viridans/A. sanguinicola/A. suis*
7	*Bacillus mycoides*	1.95	*Paenibacillus anaericanus*	70.4	*Bacillus* sp.	99.7	*B. wiedmannii/B. tropicus/B. proteolyticus*
11	*Enterococcus moraviensis*	1.83	*Enterococcus gallinarum*	85.2	*Enterococcus* sp.	100.0	*E. moraviensis/E. wangshanyuanii/E. ureilyticus*
12	*Exiguobacterium* sp.	2.19	*Bacillus halodurans*	95.2	*Exiguobacterium* sp.	100.0	*E. gingdaonense/E. algae/E. arabatum*
16	*Microbacterium maritypicum*	1.61	*Microbacterium liquefaciens*	61.0	*Microbacterium* sp.	99.7	*M. luteolum/M. algeriense/M. saperdae*
17	*Microbacterium oxydans*	2.39	*Microbacterium maritypicum*	94.8	*Microbacterium* sp.	98.4	*M. luteolum/M. algeriense/M. saperdae*
19	*Microbacterium phllosophaerae*	1.93	*Microbacterium maritypicum*	88.7	*Microbacterium* sp.	99.7	*M. luteolum/M. algeriense/M. saperdae*
18	*Microbacterium phllosophaerae*	2.01	*Microbacterium maritypicum*	82.9	*Microbacterium* sp.	99.7	*M. luteolum/M. algeriense/M. saperdae*
20	*Microbacterium phllosophaerae*	1.68	*Microbacterium maritypicum*	95.0	*Microbacterium* sp.	99.3	*M. luteolum/M. algeriense/M. saperdae*
40	*Micrococcus luteus*	2.12	*Janibacter anophelis/hoylei*	85.3	*Micrococcus* sp.	99.4	*M. luteus/M. antarcticus/M. aloeverae*
39	*Micrococcus luteus*	2.20	*Bacillus krulwichiae*	N	*Micrococcus* sp.	100.0	*M. luteus/M. antarcticus/M. aloeverae*
33	*Micrococcus luteus*	2.27	*Microbacterium maritypicum*	82.9	*Micrococcus* sp.	100.0	*M. luteus/M. antarcticus/M. aloeverae*
35	*Micrococcus luteus*	2.40	*Brevibacterium otitidis*	74.1	*Micrococcus* sp.	99.7	*M. luteus/M. antarcticus/M. aloeverae*
38	*Micrococcus luteus*	2.40	*Brevibacterium otitidis*	92.0	*Micrococcus* sp.	100.0	*M. luteus/M. antarcticus/M. aloeverae*
44	*Pseudarthrobacter oxydans*	2.39	*Janibacter anophelis/hoylei*	87.8	*Pseudarthrobacter* sp.	99.2	*P. psychrotolerans/P. phenanthrenivorans/P. nitrophenolicus*
45	*Pseudoclavibacter helveolus*	2.30	*Virgibacillus salexigens*	86.5	*Paenibacillus purispatii*	100.0	*Paenibacillus purispatii*
48	*Pseudomonas putida*	1.42	*Brachybacterium muris*	87.5	*Macrococcus* sp.	100.0	*M. canis/M. caseolyticus/M. equipercicus*
49	*Pseudomonas putida*	1.45	*Serpens flexibilis*	86.8	*Pseudomonas* sp.	99.8	*Pseudomonas* sp.
68	*Staphylococcus hominis*	2.27	*Paenibacillus provencensis*	87.0	*Staphylococcus* sp.	100.0	*S. pragensis/S. borealis/S. croceilyticus*
80	*Staphylococcus warneri*	2.00	*Staphylococcus aureus*	96.0	*Staphylococcus warneri*	100.0	*Staphylococcus warneri*
85	*Streptomyces violaceoruber*	1.38	*Paenibacillus sanguinis*	88.1	*Pseudoclavibacter terrae*	100.0	*Pseudoclavibacter terrae*

N—identification probability not available. * A MALDI score of >2.0 indicates a reliable identification at the species level; a MALDI score between 1.70 and 1.99 indicates a reliable identification at the genus level. ^§^ A match of ≥99.0% or ≥97.0% is considered to belong to the same species or genus, respectively.

**Table 4:** Number of raw reads per sample and the percentage of reads that can be assigned to a specific higher taxonomic group of microorganisms.

Sampling Site	Sample Type	Number of Raw Reads	Microbial Reads (%)	Bacterial Reads (%)	Viral Reads (%)	Fungal Reads (%)	Protozoan Reads (%)
Jedilnica before therapy	Nitrocellulose filter	5,978,569	80.2	75.9	0.00095	0.00184	0.00314
Jedilnica before therapy	Collection liquid	6,950,426	36.7	31.2	0.00099	0.00302	0.00119
Jedilnica before therapy	Collection liquid duplicate	4,666,878	86.9	85.3	0.00024	0.30500	0.10400
Jedilnica after therapy	Nitrocellulose filter	2,323,706	84.2	79.9	0.00039	0.00435	0.00409
Jedilnica after therapy	Collection liquid	6,293,127	8.35	0.8	3.3	0.00663	0.00923
Jedilnica after therapy	Collection liquid duplicate	13,148,700	71.7	17.3	50.7	0.42200	0.00294
Spalnica before therapy	Nitrocellulose filter	5,665,593	94.7	94.3	0.00005	0.04660	0.00007
Spalnica before therapy	Collection liquid	4,509,290	17.5	14.0	0.01510	0.03830	0.01590
Spalnica before therapy	Collection liquid duplicate	27,049,966	41.9	36.2	0.01910	0.46200	0.02200
Spalnica after therapy	Nitrocellulose filter	5,707,536	83.4	72.5	7.7	1.1	0.00137
Spalnica after therapy	Collection liquid	3,274,666	50.9	0.09	45.8	0.00083	0.00018
Spalnica after therapy	Collection liquid duplicate	6,049,608	54.2	28.7	25.0	0.02690	0.00063
Telovadnica before therapy	Nitrocellulose filter	7,298,271	80.4	78.9	0.00058	0.00174	0.0037
Telovadnica before therapy	Collection liquid	117,357	27.7	26.7	0.00682	0.08180	0.00170
Telovadnica before therapy	Collection liquid duplicate	8,019,772	71.3	68.2	0.03170	0.03250	0.00168
Telovadnica after therapy	Nitrocellulose filter	8,963,828	76.0	31.5	39.9	0.02200	0.00167
Telovadnica after therapy	Collection liquid	8,976,140	4.07	0.771	2.5	0.00136	0.00031
Telovadnica after therapy	Collection liquid duplicate	8,947,973	52.1	51.2	0.00834	0.1	0.00750

## Data Availability

Data are contained within this article.
